# A Versatile Method to Produce Monomodal Nano‐ to Micro‐Fiber Fragments as Fillers for Biofabrication

**DOI:** 10.1002/smtd.202401060

**Published:** 2024-12-17

**Authors:** Zan Lamberger, Vivien Priebe, Matthias Ryma, Gregor Lang

**Affiliations:** ^1^ Department for Functional Materials in Medicine and Dentistry University Hospital of Würzburg Pleicherwall 2 D‐97070 Würzburg Germany

**Keywords:** cryo‐cutting, electrospinning, fiber fragments, fiber‐spinning, MEW

## Abstract

A key goal of biofabrication is the production of 3D tissue models with biomimetic properties. In natural tissues, fibrils—mainly composed of collagen—play a critical role in stabilizing and spatially organizing the extracellular matrix. To use biomimetic fibers for reinforcing bioinks in 3D printing, fiber fragmentation is necessary to prevent nozzle clogging. However, existing fragmentation methods are often material‐specific, poorly scalable, and provide limited control over fragment size and shape. A novel workflow is introduced for producing fiber fragments applicable to various materials and fabrication techniques such as electrospinning, melt‐electrowriting, fused deposition modeling, wet spinning, and microfluidic spinning. The method uses a sacrificial membrane as a substrate for precise cryo‐sectioning of fibers. A significant advantage is that no additional handling steps, such as fiber detachment or transfer, are needed, resulting in highly reproducible fiber sectioning with a quasi‐monodisperse length distribution. The membrane can be rolled before cutting, preventing fibers from sticking together and significantly increasing production efficiency. This method is also versatile, applicable to multiple fiber types and materials without re‐parameterization. Cell culture experiments demonstrate that the fibers maintain key properties necessary for cell‐fiber interactions, making them suitable for systematic screenings in the development of anisotropic 3D tissue models.

## Graphical abstract

This research introduces an innovative approach for efficiently generating fiber fragments as additives for 3D‐biofabrication. Utilizing a sacrificial membrane, the method enables highly reproducible, scalable, and precise cryo‐cutting of fibers, ensuring compatibility across diverse materials and processes like electro‐ and microfluidic spinning, melt‐electrowriting, and fused deposition modeling. Cell‐culture experiments validate the fibers' efficacy in facilitating systematic studies of cell‐fiber interactions.

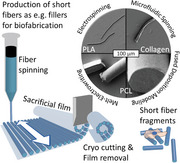

## 1 Introduction

In the natural extracellular matrix (ECM), fibrous proteins, with collagen I being the most predominant, provide structural integrity to the water‐based hydrogel matrix while also acting as a cell‐guiding structure.^[^ ^1^, ^2^ ^]^ Biofabrication aims is to mimic such matrices, whether as part of organs or tissues.^[^ ^3^ ^]^ Thus, to recapitulate the reinforcing and cell‐guiding effect of collagen, fibrous substrates are often used as substitutes. In 2D models, this is usually in the form of electrospun membranes with random or aligned fibers made from synthetic or natural materials,^[^ ^4^, ^5^ ^]^ whereby the nano‐ or micrometer‐sized continuous fibers primarily serve to mimic the role of fibrillar ECM proteins as guiding structures.^[^ ^6^ ^]^ When implemented in 3D cultures, membranes may be stacked or spun into foam‐like meshes filled with hydrogel,^[^ ^7^ ^]^ thereby giving the fibrous substrates the additional role of mechanically stabilizing the hydrogel. Similarly, larger melt‐electrowritten (MEW) substrates can be used to stabilize soft hydrogels while simultaneously modulating cell shapes.^[^ ^8^, ^9^ ^]^

While such substrates can be implemented if the hydrogels are cast or deposited independently of the fibers, limitations arise when fabricating complex geometries or trying to achieve high resolutions. For such applications, bioprinting is usually employed, as it enables precise deposition of the hydrogel matrix and cells in the form of a printable bioink.^[^ ^10^ ^]^ While much work has been done on the hydrogel and cell aspects of such bioinks, mechanical stability, and cell anisotropy remain highly desired.^[^ ^11^ ^]^ This may be achieved by incorporating fibrous structures into the bioinks used in extrusion bioprinting.^[^ ^12^, ^13^ ^]^ The fibers in such bioinks must be reduced to short lengths, as continuous or very long fibers cannot be printed due to issues of aggregation and clogging of the bioprinter nozzle.^[^ ^12^, ^14^ ^]^ If the fibers have an adequate length, they can then be aligned by the shear and elongational flow that occurs during extrusion through the nozzle, facilitating fiber alignment with the printed strut direction.^[^ ^14^, ^15^ ^]^ These fibers can then act as guiding structures for directing cell migration and alignment of the tissue, which is especially relevant for skeletal muscle,^[^ ^16^ ^]^ heart muscle,^[^ ^17^, ^18^ ^]^ and nerve tissues.^[^ ^19^, ^20^ ^]^ Furthermore, fibers used as filler materials have been shown to modulate the rheological and mechanical properties of materials.^[^ ^12^, ^13^, ^21^, ^22^ ^]^ Hence, especially when combined with typically soft or non‐printable hydrogel‐based materials, fibers can provide a strengthening role, potentially offering an alternative to overcome the biofabrication window, which defines the impasse between bioink resolution/stability and cell proliferation.^[^ ^11^ ^]^ Moreover, by using fiber gradients or functionalized fibers, they can be utilized to more effectively manipulate mechanical properties and cell‐guiding effects,^[^ ^23^ ^]^ or modified with different factors that can be controllably released to further fine‐tune cellular behavior.^[^ ^24^, ^25^ ^]^

Nonetheless, there are only a few examples of successfully implementing fibers in bioinks^[^ ^14^ ^]^ or biomaterial inks.^[^ ^21^ ^]^ The main challenge here is finding the appropriate fiber type for the application and then managing to produce cut fibers of adequate size and in sufficient quantities. To ascertain that fibers perform best for their desired effects, many different diameters, lengths, morphologies, materials, etc., must be tested. However, producing and cutting/fragmenting these fibers is often problematic, as it involves different spinning methods and fiber characteristics, requiring laborious adaptations of the cutting/fragmenting procedures, which can yield mixed or incomparable results, sometimes even damaging the fibers.^[^ ^26^ ^]^ Hence, there is an immense lack of literature concerning the printability of different cut fibers and their impact on cells, providing scientists in the field with very little starting information when trying to develop their complex tissue models. In general, fiber fragmentation technologies can be categorized into two primary methodologies: in situ techniques, which modify fibers during their formation, and post‐spinning techniques, which alter fibers after they have been produced. In situ fragmentation manipulates solution and spinning parameters to control fiber lengths as they are formed. One example is the Xanoshear process, an industrial technique that produces polylactic acid (PLA) fibers with an average length of 67 µm and a significant variability (±49 µm).^[^ ^27^ ^]^ Another in situ method involves electric spark‐assisted electrospinning, where a polymer solution in an organic solvent is extruded from a syringe needle and passes through an electric spark gap before adhering to a collector plate. The fragmentation occurs due to the spark, resulting in fibers ranging from 22 to 400 µm in length.^[^ ^28^ ^]^ A further example is the entanglement loss‐assisted breakage mechanism, which allows for the adjustment of fiber lengths by varying the flow rate of the polymer solution and the applied voltage during electrospinning. Higher flow rates yield longer fibers, while increased voltage results in shorter fibers, producing microfibers that range from 37 to 670 µm.^[^ ^29^ ^]^ Additives such as silica particles can be incorporated to further reduce the fiber length.^[^ ^30^ ^]^ Another in‐situ fragmentation method is entropy‐elastically driven fiber snapping, which occurs during the drying of a solution electrospun polycaprolactone (PCL) fiber but can also be induced during cooling a MEWCL strand.^[^ ^31^, ^32^, ^33^ ^]^

Post‐spinning fragmentation refers to techniques applied to fibers after their initial formation. Mechanical fracturing methods such as cutting, grinding, and homogenization are common post‐spinning methods. For example, aligned fiber mats can be produced by spinning on a rotating drum and subsequent cutting with razor blades under liquid nitrogen yields in rod‐like non‐aggregated fibers with an average length centered ≈50–100 µm for polymethyl methacrylate (PMMA) and 50–500 µm for polyimide fibers.^[^ ^34^, ^35^, ^36^ ^]^ Besides the relatively broad fiber diameter distribution, this method is only partially efficient as it is difficult to scale. In contrast, scalable methods such as rubber milling^[^ ^37^ ^]^ and cryogenic milling^[^ ^38^ ^]^ have been conducted for the scission of electrospun membranes. However, these methods offer limited control over the fiber length distribution and may alter the morphology due to heat generation and the strong mechanical impact. Shear stress‐based methods also play a role in post‐spinning fragmentation. For example, fibers can be suspended and then fragmented by nozzle extrusion under high shear stress, often resulting in fibers shorter than 20 µm.^[^ ^39^ ^]^ Comparable results (≈10 µm in length for polystyrene and PMMA) were also achieved through the ultrasonication of electrospun membranes.^[^ ^40^, ^41^ ^]^ In another approach, fibers dispersed in toluene/petroleum mixtures are subjected to intense stirring, producing an average fiber length of 220 ± 112 µm.^[^ ^42^ ^]^ Moreover, various approaches have utilized homogenization to break down nano‐ and microfibers, resulting in a polydisperse fiber length distribution within the sub‐100 µm range.^[^ ^43^, ^44^, ^45^ ^]^ Chemical treatment and UV irradiation are additional post‐spinning methods that offer control over fiber length. For instance, aminolysis‐induced degradation and crystallization was performed to fabricate aminated PLA and poly L lactic acid (PLLA) nanocylinders from electrospun nanofibers.^[^ ^46^, ^47^, ^48^ ^]^ Alternatively, UV light can be used to cut fibers through a mask with adjustable slits, tailoring fiber lengths according to the mask design.^[^ ^49^ ^]^ Nevertheless, these techniques can potentially have a significant impact on morphological and chemical integrity of the fibers.

While these methods have generally been successful in producing fiber fragments, the range of materials, lengths, and different fiber morphologies that can be produced with only slight adaptations of the methods are limited. An alternative, material‐independent method is cryo‐cutting using devices like microtomes, whereby an embedded frozen fibrous substrate can be cut to size using a precision blade.^[^ ^50^ ^]^ While initially this approach was constrained to thick, stiff, aligned electrospun membranes, the stability of these was often compromised by the collection, transfer, and embedding in cryo‐liquid, which could misalign the fibers and deform the sample.

**Figure 1:**
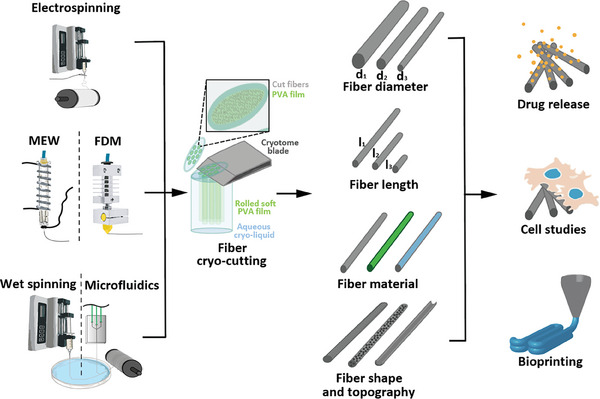
Overview of the various fiber production methods used in this work. All fiber types were processed into fragments using the same cryo‐cutting‐based workflow. This enables the production of fiber fragments with controlled diameter and length from different materials, independent of fiber topography, using a single method. The fibers have the potential to be loaded with bio‐active substances, and specific cell‐fiber interactions with different fiber types can be studied. Due to the limited fiber length, they are also well‐suited as fillers in the field of bioprinting.

We solved the issue of producing precisely cut fibers by combining the cryo‐cutting method with a soft, adhesive, water‐soluble film that can be implemented with any fiber production method, acting as a substrate onto which the fibers can be collected in an aligned manner, easily transferred, and cryo‐cut. After the removal of the water‐soluble components, this results in low polydispersity fibers of desired lengths, independent of material, morphology, or fiber spinning method, with good yields and easily scalable production. Thus, this approach enables quick production and adaptations when tailoring different cut fibers for direct comparison in cell culture and screening in bioprinting applications, as schematically shown in **Figure** **1**.

## 2 Results and Discussion

To cut fibers to a defined length, they must be well‐aligned, which can be achieved in most processes using rotating collectors (in spinning processes) or parallel deposition (in printing or MEW). However, the critical step is the transfer, where the fibers need to be moved to the cryo‐cutter. In the past, there has been no standardized procedure available that yields reproducible results, as it heavily depends on the user's handling skills. In this work, a versatile workflow was developed to fabricate reproducible and scalable fiber fragments with precisely adjustable lengths.

### 2.1 General Workflow

**Figure 2:**
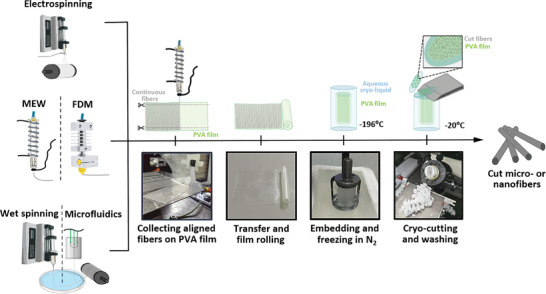
Schematic depiction of the processing for fiber cutting. The fibers are spun or printed onto soft, adhesive, water‐soluble PVA‐based films, which can be rolled up, embedded in cryo‐cutting fluid, frozen in liquid nitrogen, and precisely cut to the desired size using the cryotome. The PVA is then removed by washing in 70% ethanol or other aqueous media, resulting in purified cut fibers of the desired length, as set by the cryotome.

The preparation of cut fibers in this workflow involves producing fibers using various spinning procedures, such as electrospinning, MEW, fused deposition modeling (FDM) 3D printing, wet spinning, and microfluidic spinning. The fibers are deposited onto an adhesive, soft polyvinyl alcohol (PVA)‐based film, which facilitates their transfer and easy embedding in the cryo‐liquid, specifically Tissue‐Tek O.C.T. compound. The fibers on the film can be frozen in liquid nitrogen, cryo‐cut to the desired size using a commercially available cryotome, and purified to obtain the desired cut fibers without any residues and with low fiber length polydispersity, thereby eliminating the need for further size‐based purification, as shown in **Figure** **2**. This method can be easily adapted to various fiber spinning procedures and most fiber materials. Thus, reproducible and scalable fiber fragments with precisely adjustable lengths can be fabricated in a standardized manner.

### 2.2 Optimization of Film Properties

To make the sacrificial membrane suitable as a substrate for fiber transfer in as many processes as possible, the crucial design criteria were defined initially:

- Good solubility under moderate conditions: PVA is particularly suitable because it is soluble in water and up to 70% ethanol, which is often used for sample sterilization.
- Mechanical stability and flexibility: The films must be stable and deformable enough to avoid cracking, allowing them to be wrapped around a cylindrical collector.
- Self‐adhesion: Ideally, the films should exhibit strong self‐adhesion when rolled up, preventing the collected fiber bundles from unrolling during handling.
- Fiber immobilization: The films’ stickiness must keep fibers immobilized during collection and processing. This is especially useful in FDM printing, where adhesion of the printed strut is crucial but can be challenging on unheated substrates.

**Figure 3:**
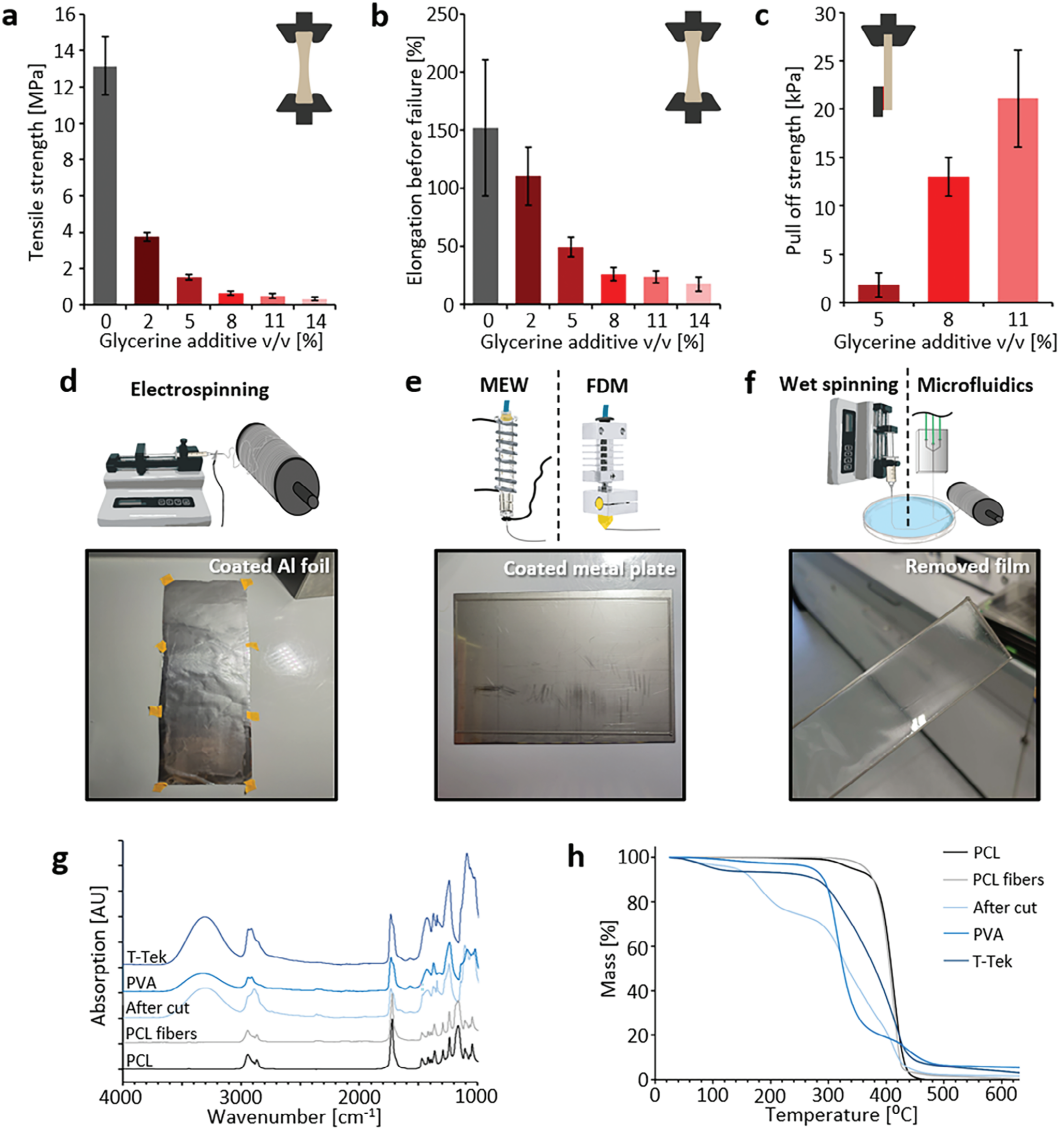
Properties of soluble films and film removal during fiber purification. a) Tensile testing of PVA films with increasing glycerin shows yield stress, and b) elongation before failure. c) Pull‐off tests with increasing glycerin concentration demonstrate increased adhesion. PVA films can be cast onto various substrates for different fiber production techniques: d) aluminum foil for electrospinning, e) metal plates for MEW and FDM printing, and f) a free‐standing film supported by a frame, which can be reattached to a rotating collector due to its adhesive forces. g) Fourier transform infrared spectroscopy (FTIR) spectra show the removal of Tissue‐Tek and PVA from cut PCL fibers after the washing steps, as indicated by the stepwise decrease of the PVA and glycol‐characteristic broad O–H stretching band (3685–3010 cm^−1^). h) thermogravimetric analysis (TGA) measurements of the samples measured before, during, and after washing. Values in panels a), b), and c) are mean ± standard deviation, n = 9.

Based on preliminary tests, it was found that pure PVA films exhibit low fiber adhesion and are too stiff and brittle for some applications. Therefore, glycerin was added as a plasticizer, since it is cheap and easy to handle, soluble in water/ethanol mixtures, and biocompatible, while it also imparted adhesive properties to the films. Glycerin was added various concentrations, and the mechanical properties were measured, as shown in **Figures** **3**a–c and S1a,b (Supporting Information). It was observed that both tensile strength and elongation significantly decreased with increasing glycerin concentration, as seen in Figure 3a,b, while adhesiveness increased markedly, as shown in Figure 3c. Films with a higher glycerin concentration may have an even higher adhesion, these were too fragile to handle and would easily tare, making removal of the films from the substrate too difficult, thus making them disadvantageous. Furthermore, even at low concentrations of 2% v/v, there was a significant decrease in the elastic modulus, indicating that the bending stiffness of the films also decreases, making them easier to wrap, as demonstrated in Figure S1c (Supporting Information). Since both adhesiveness and wrappability were identified as key factors in the suitability of the films, subsequent work was carried out with 11% v/v glycerin, which proved to be the best compromise in practical tests.

Due to its malleability and adhesiveness, the same film can be employed in several variations, covering the needs of most conventional fiber‐producing procedures. For instance, in electrospinning, the film could be cast atop aluminum foil that is then wrapped around the collector, as shown in Figure 3d. For MEW and FDM, the film could be cast atop metal plates that were then placed onto the machine's build plate, as in Figure 3e. Alternatively, the film could also be removed from these plates before collecting, as demonstrated in Figure 3f, and wrapped around smaller rollers for wet spinning/microfluidic spinning or placed onto any other collecting substrate.

### 2.3 Removal of the PVA and Cryo‐Cutting Liquid

After being cut, fiber purification is essential, especially if the fibers are intended for use with sensitive eukaryotic cells, incorporation into bioinks, or potential implantation into animals or humans. Therefore, any substances that could cause cytotoxicity or inflammation must be removed. PVA is an FDA‐approved material and is not cytotoxic, while the Tissue‐Tek O.C.T. compound, used to stabilize and hold the sample in place during cryo‐cutting, consists of a water‐soluble mixture predominantly made up of glycerin, PVA, and other non‐reactive glycols and resins, as stated by the manufacturer. These water‐soluble polymers are removed by washing the sample after cutting in aqueous media and up to 70% ethanol. For non‐proteinaceous fibers, 70% ethanol is typically used due to its lower density, which facilitates fiber sedimentation during washing. If fibers do not sediment in the solution, the solution is filtered off. Phosphate‐buffered saline (PBS) can be used when processing proteinaceous fibers such as gelatin or collagen, which are prone to aggregation and denaturation when exposed to ethanol solution.^[^ ^51^, ^52^ ^]^

To demonstrate the removal of PVA and Tissue‐Tek, MEW‐spun PCL fibers were used. In FTIR analysis, the PCL signal is distinct from the PVA and Tissue‐Tek signals in the O–H stretching band (3685–3010 cm^−1^), which is characteristic of PVA and glycols.^[^ ^53^ ^]^ The removal of PVA and Tissue‐Tek could be visualized in Figure 3g, showing the complete disappearance of the O‐H signal for the cut fiber sample after the washing steps, closely resembling the signal of the unprocessed PCL pellet. This is also the case for PCL fibers produced using electrospinning and FDM, as shown in Figure S1e (Supporting Information).

Additionally, TGA analysis was used to assess whether any residual polymer besides PCL remained in the sample after washing, as shown in Figure 3h. The thermal degradation curve for the fibers was identical to that of the PCL pellet from which they were made. There is a slight deviation between the two curves, likely due to the higher surface area of the fibers compared to the pellet, which appears to result in more homogeneous degradation. In any case, these curves showed no trace of PVA or Tissue‐Tek residues, which degrade at lower temperatures than PCL. No measurable traces of PVA or Tissue‐Tek were found, and even if these substances were still present at concentrations below the detection limit, both are non‐cytotoxic.

### 2.4 Application of the Workflow for Different Processes

#### 2.4.1 Electrospinning and Fiber Fragmentation

**Figure 4:**
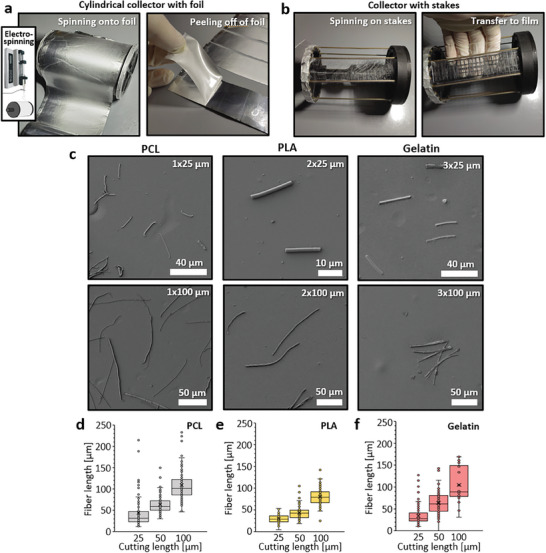
Processing of electrospun fibers of different materials with the cryo‐cutting method. Two alternatives for collecting electrospun fibers are presented: a) fibers can be collected on a collector covered with aluminum foil coated with the PVA/glycerin film, which can be cut and separated from the foil after spinning (as applied for PCL and PLA), or b) fibers can be collected between stakes and then transferred to the adhesive PVA/glycerin film after spinning (as applied for gelatin). c) Representative images show fibers of PCL, PLA, and gelatin cut to different lengths, along with graphs depicting the length distribution of the fibers for the preset cutting lengths (d‐f). The plotted values in d, e, and f are presented as mean ± standard deviation, with fiber diameters indicated as average (x) and median (–). Number of measurements are as follows: d, n = 100; e, n = 100; f, 25 µm, n = 100; 50 µm, n = 100; 100 µm, n = 30.

To make the method suitable for electrospun fibers, a PVA/glycerin film was initially cast on aluminum foil, which was then fixed onto a cylindrical collector. At high speeds, aligned fibers made from various materials (PCL and PLA) were spun onto the film, which was subsequently peeled off and cut into strips at a right angle to the fiber orientation, as shown in **Figure** **4**a. Alternatively, free‐hanging fibers, such as those on parallel bars, can also be removed using PVA/glycerin films (Figure 4b). This second method was applied to gelatin fibers, which were subsequently crosslinked via glutaraldehyde vapor while still attached to the film, demonstrating the potential of the film for use during such non‐aqueous post‐processes. In the final step, the strips were wound up and cut to the desired length in the cryo‐cutter before the PVA/glycerin was removed to obtain pure fiber fragments. Scanning electron microscopy examination revealed homogeneous fibers for all tested materials, with no artifacts such as film residues or damage visible in Figure 4c, whilst fiber morphology and diameters before and after cutting were also retained, as shown in Figure S2b,c (Supporting Information). Furthermore, quantitative analysis demonstrated excellent control over fiber lengths with low length distribution, as can be seen in Figure 4c. Therefore, the method not only consistently works for very small fiber diameters but is also independent of the material used to produce the fibers. This versatility allows for harnessing the potential of various materials in novel research and applications using small‐cut fibers. Since electrospinning, in particular, is a straightforward process for producing fibers with diameters in the nano‐ to micrometer range,^[^ ^54^, ^55^ ^]^ the method is highly suitable for producing biomimetic fibers that mimic the size range of collagen fibers in the ECM. Thus, it is relevant for a broad variety of applications in the field of tissue engineering and biofabrication.^[^ ^56^ ^]^ Moreover, our method addresses the recently formulated demand for material‐independent fragmentation techniques with sufficient yield^[^ ^26^ ^]^ and simplifies the handling of fragile samples prone to fusion and agglomeration.^[^ ^31^, ^57^ ^]^

#### 2.4.2 MEW and Fiber Fragmentation

**Figure 5:**
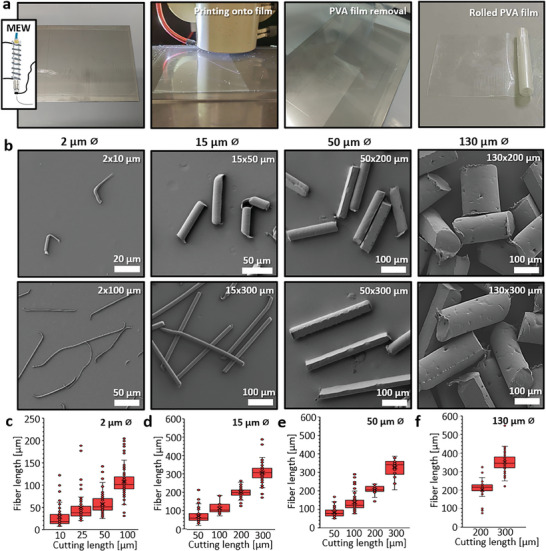
Processing of MEW PCL fibers of various diameters into different lengths. a) A metal plate coated with a soft PVA film, onto which aligned fibers are deposited using MEW. The PVA film is then cut into smaller strips, pulled off the plate, and rolled up for embedding and cryo‐cutting. b) Exemplary images of MEW PCL fibers with different diameters, cut to various lengths using the described cutting technique. The corresponding measured fiber lengths are compared to the cutting settings for fibers with approximate diameters of c) 2 µm, d) 15 µm, e) 50 µm, and f) 150 µm. Plotted values in c, d, e, and f are presented as mean ± standard deviation of fiber diameters: c) 10 µm n = 60, 25 µm n = 70, 50 µm n = 100, 100 µm n = 100; d) 50 µm n = 66, 100 µm n = 31, 200 µm n = 46, 300 µm n = 87; e) 50 µm n = 72, 100 µm n = 74, 200 µm n = 17, 300 µm n = 25; f) n = 75.

To cut MEW fibers to a defined length after printing, the metal collector plate of the MEW device was coated with a PVA/glycerin film. This coated plate was then placed onto the build plate of the MEW device, where the fibers were collected, as shown in **Figure** **5**a. After printing, the film can be cut into smaller strips perpendicular to the printing pattern, peeled off the plate, and rolled up for cutting, as demonstrated in Figure S3a (Supporting Information). This method makes the entire collection and transfer process significantly easier compared to the process without using the film, as indicated in Figure S3b–d (Supporting Information).

Using MEW, the diameter of the PCL fibers can easily be adjusted from 2 to 130 µm or more, allowing for the rapid production of fibers with different sizes. Fibers with diameters of 2, 15, 50, and 130 µm were used to demonstrate the method's ability to collect, transfer, and cut fibers of varying diameters, as plotted in Figure S3e (Supporting Information). These fibers were then cut into lengths ranging from a minimum of 10 µm to a maximum of 300 µm. After the PVA/glycerin was removed by washing with 70% ethanol, the cut fibers were visualized via scanning electron microscopy (SEM), as shown in Figure 5b. The quantification of fiber length revealed a strong correlation between the expected and actual fiber lengths, as plotted in Figure 5c–f, with most fibers in each batch showing a very narrow length distribution. However, for very short lengths and small cutting intervals, such as with the 2 µm fibers shown in Figure 5c, some protrusions were observed due to the stretching of the fragile PCL fibers. Nevertheless, apart from the ends, the diameters and morphology of the MEW fibers were generally preserved, as compared to the samples before cutting, as shown in Figure S3f (Supporting Information).

To scale up the production of fibers on one sheet, several layers can be printed. Adhesion between the deposited layers is inhibited by spraying each layer with a 1:2 (v/v) solution of 20% (w/v) PVA in 70% ethanol. The yield of the procedure depends on the type of fiber, the collection method, and the purification process used, with the limiting factor for production quantity being the duration of fiber production rather than the cutting and post‐processing. To exemplify the yield, a 15 µm diameter and 100 µm long PCL fiber was produced using MEW in one layer and five layers to demonstrate vertical scalability. The deposited weight of one layer under the given production conditions was 7 mg. After cutting and purification, 4.8 mg of the one‐layer fiber and 26.1 mg of the five‐layer fiber were obtained, resulting in yields of 69% and 74%, respectively.

MEW can be used to produce larger diameter fibers, which are usually difficult to produce using electrospinning, ranging from a few µm to 100 µm or more. Scaffolds produced by this method are already widely used to investigate cellular behavior,^[^ ^9^, ^58^, ^59^ ^]^ with fiber diameters or other properties being of great interest for how they impact cellular interactions. The use of larger diameters is also being considered for filler materials in bioinks to guide cells and modulate rheological properties.^[^ ^12^, ^13^, ^21^, ^60^ ^]^ However, one of the main limitations is that with regular MEW onto a collector, producing aligned fibers and cryo‐cutting them into small fiber fragments has been difficult due to transfer issues and misshaping, as shown in Figure S3a,b (Supporting Information). While some methods for producing fiber fragments exist, such as fiber snapping,^[^ ^32^ ^]^ these are likely not transferable to other materials, and the gaps and printing parameters must be fine‐tuned for each fiber diameter. By using the cryo‐cutting method presented here, combined with the PVA/glycerin film as a substrate, previously problematic aligned fibers could be collected, transferred while retaining their alignment, and precisely cut.

#### 2.4.3 Microfluidic Wet Spinning and Fiber Fragmentation

Wet spinning is another commonly used method to produce fibers, where the fiber precursor solution is extruded from a syringe into a bath of antisolvent, causing the polymer to precipitate and form a fiber.^[^ ^61^, ^62^, ^63^ ^]^ This stable fiber is typically removed from the solution and collected on an external collector. The process can be further refined by using a microfluidic chip to produce even smaller fibers than what is achievable with a regular syringe tip.^[^ ^62^, ^64^ ^]^ If the fiber needs to be cut after processing, it often has to be unrolled from the collector or cut while still on it, leading to difficulties, particularly when short fiber lengths are required.

**Figure 6:**
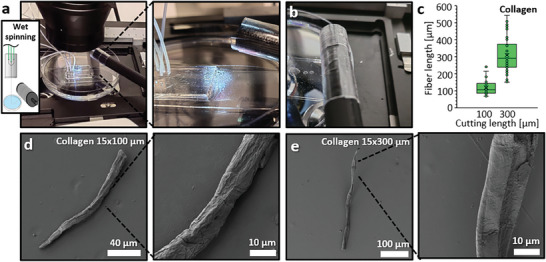
Cutting of microfluidically wet‐spun collagen fibers to different lengths. a) The setup for microfluidically spinning a collagen I fiber, illustrating the fiber being collected. b) The collection was done onto a roller wrapped with a PVA film. c) Graph showing the distribution of two different lengths to which the collagen fiber was cut, and (d and e) representative images of these fibers. Plotted values in c) are presented as mean ± standard deviation of fiber diameters, with x as the average and – as the median, n = 36 and n = 50.

We utilized microfluidic wet spinning of a collagen I fiber, as shown in **Figure** **6**a, to demonstrate the improved collection and cutting procedure when employing the PVA film. In this case, the film was wrapped around the roller, where the fiber was collected after being pulled from the aqueous precipitation solution, as shown in Figure 6a,b. Despite the film's water‐soluble properties, this did not pose an issue, as the amount of water carried out with the fiber was minimal. After collection, the film was cut in half and removed from the collector before undergoing cryo‐cutting. This resulted in collagen fibers cut to ≈100 and 300 µm in length, with diameters of ≈17 µm, as shown in Figure 6c and Figure S4a (Supporting Information). The fibers exhibited a satisfactory size distribution, as plotted in Figure 6d,e.

It is worth noting that in cases where wet spinning is conducted in non‐aqueous solvents, collection can be done directly in the precipitation bath, as PVA is insoluble in most solvents except water.^[^ ^65^ ^]^ Additionally, if fibers are too fragile to be pulled from an aqueous precipitation bath, they can be collected onto a polyethylene (PE) foil‐covered collector. After removal, the PE foil can be bonded to the PVA film, allowing the PE foil to be peeled away, leaving the fibers adhered to the more adhesive PVA film. Once transferred, the fibers can again be cut using the same method described throughout the work.

#### 2.4.4 FDM 3D Printing and Fiber Fragmentation

**Figure 7:**
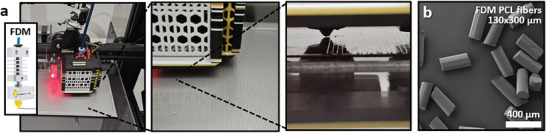
Collecting and cutting FDM printed PCL fibers. a) FDM printing of aligned PCL fibers onto a PVA film on an unheated metal plate and b) an example of the resulting cut fibers.

A method for producing fibers involves extruding the melt through a nozzle and allowing it to cool.^[^ ^66^ ^]^ When done in a free‐floating setup, the polymer strand can be easily collected onto a cylindrical collector covered with PVA foil, provided the fibers are soft enough. However, if stiffer fibers are produced, bending and manipulation after collection may become challenging. The solution is to deposit these fibers on a flat surface, similar to the process shown in the section on MEW (Figure 5a). In this case, the PVA film again plays a crucial role by enabling not only easy transfer of the deposited aligned fibers but also much better deposition. The film's strong adhesion makes heating the build plate unnecessary, as shown in **Figure** **7**a. Additionally, the PVA film offers excellent adhesion during printing and allows for easy removal afterward, unlike direct deposition onto the build plate, where printed strands are often difficult to remove without damage due to excessive adhesion or require heating to print effectively. Using this method, PCL fibers were extruded through a 0.1 mm nozzle onto a PVA film‐covered metal plate in an aligned pattern and processed similarly to MEW samples. The resulting 130 µm diameter fibers exhibited a good size distribution and morphology, as shown in Figure 7b and Figure S4b (Supporting Information), comparable to MEW fibers. The key difference is that using FDM printing allows for faster and more reliable production of larger fibers than MEW, which is better suited for fibers with diameters below 100 µm.^[^ ^67^ ^]^

### 2.5 Cell Culture Investigation of Different Cut Fibers

**Figure 8:**
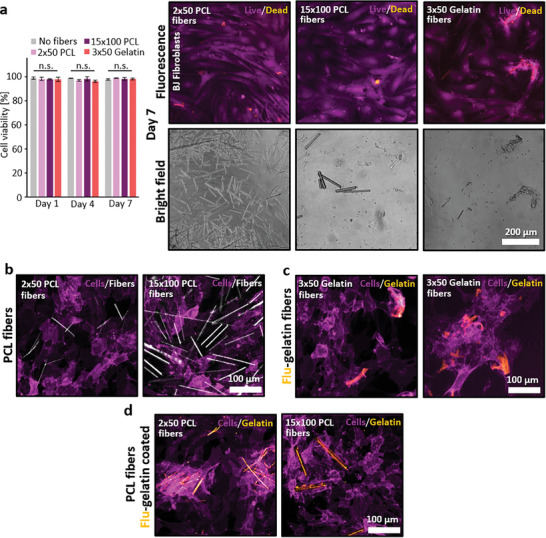
Culture of BJ‐TERT human fibroblast cells with different cut fibers to screen for negative effects and cell‐fiber interactions. a) Live‐dead analysis of cells cultured without fibers or with 2 × 50 and 15 × 100 µm PCL fibers, as well as 3 × 50 µm cut gelatin fibers. Representative fluorescence and bright field images are shown from day 7, illustrating the presence of the fibers. b) Confocal images of autofluorescent td‐Tomato labeled BJ‐TERT fibroblasts alongside uncoated 2 × 50 and 15 × 100 µm PCL fibers. c) Images of autofluorescent fibroblasts interacting with 3 × 50 µm fluorescently labeled gelatin fibers. d) Autofluorescent fibroblasts with fluorescently labeled gelatin‐coated 2 × 50 and 15 × 100 µm PCL fibers after 1 day of culture. Plotted values in a) represent mean ± cell viability (n = 3). P‐values were calculated using a two‐way ANOVA, with significance indicated as ^*^P < 0.05.

The cutting method simplifies the production and fragmentation of various fibers, enabling systematic screening of cell‐fiber interactions that vary depending on the materials, coatings, or fiber morphologies. To exemplify this, BJ‐TERT human fibroblast cells were exposed to PCL fibers measuring 2 × 50 and 15 × 100 µm, as well as gelatin fibers of 3 × 50 µm, with the live/dead results displayed in **Figure** **8**a. To minimize the effects of fiber attractiveness and size, the cells were plated onto a treated surface well plate, which promotes preferential adhesion and proliferation, regardless of the fiber presence. Even with a substantial amount of fibers in the well, no significant negative effects on cell viability were observed after a period of 7 days, nor did dead cells colocalize with the fibers.

As reported in other literature, the fibers were not inherently harmful to the cells; however, the cells showed a reluctance to interact with the PCL fibers.^[^ ^13^, ^31^, ^68^ ^]^ This was effectively visualized using confocal microscopy, which captured the fibers and the autofluorescent BJ‐TERT TdTomato‐farnesyl expressing cells^[^ ^69^ ^]^ simultaneously, as shown in Figure 8b. The images revealed that the cells appeared to grow undisturbed by the fibers, likely avoiding them and proliferating beneath the larger fibers due to the more attractive treated surface of the well. In this case, the thicker fibers appeared thinner due to the refractive imaging used for visualization, as the core fluoresced more prominently than the outer layers.

The cells demonstrated a greater tendency to contact and cluster around the fluorescently labeled gelatin fibers compared to the PCL fibers, as evident from Figure 8c. This behavior is attributed to gelatin's inherent cell‐attractive properties, similar to collagen,^[^ ^70^, ^71^ ^]^ whereas the attractiveness of PCL fibers is primarily influenced by what is adsorbed or bound to their surface.^[^ ^31^, ^72^, ^73^ ^]^ Since cells migrate and adhere more effectively to surfaces that are preferentially attractive,^[^ ^74^, ^75^, ^76^ ^]^ it is not surprising that they prefer to interact with gelatin fibers or the treated well plate over the PCL fibers. To further illustrate this point, when cells were placed on an agarose well surface alongside PCL fibers, they began to interact and cluster around the fibers. In this scenario, the PCL fibers provided better adhesion than the cell‐repellent agarose surface, as shown in the exemplary image in Figure S5b (Supporting Information).

To enhance the attractiveness of the PCL fibers, they were further coated with gelatin and labeled with NHS‐fluorescein, which made the coating fluoresce. This coating was successful, as the gelatin fluorescence was visible under the microscope. The coated fibers, depicted in Figure 8d, became more attractive, leading the cells to interact with them and form clusters around the fibers. This effect was particularly pronounced with the 15 × 100 µm fibers, where the cells wrapped around the fibers rather than ignoring them or growing underneath. This interaction was clearly visible in Figure S5c (Supporting Information), where the fluorescence of the fibers was reduced in the image, allowing the fibers to be seen more as cavities.

After the fiber cutting and processing, the fibers remained fully suitable for cell culture applications, showing no signs of cytotoxicity or harmful residues. Furthermore, they can be modified with proteinaceous or other coatings to enhance their attractiveness to cells. This also exemplifies the varying effects of materials, coatings, and sizes on cellular behavior, highlighting the importance of precise comparisons in this field.

### 2.6 Exemplary Bioprinting of Fibers

**Figure 9:**
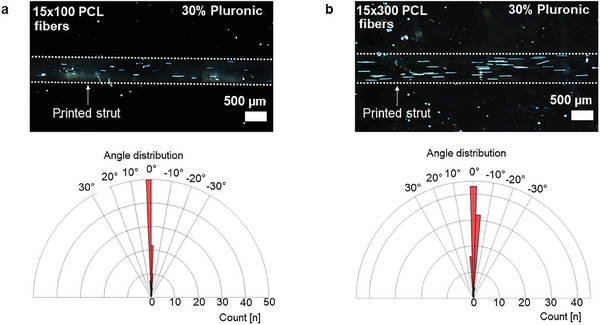
a) Extrusion‐printed 15 × 100 µm PCL fibers aligned in a 30% (w/v) Pluronic ink. b) Aligned 15 × 300 µm PCL fibers printed in the same ink, accompanied by the corresponding alignment graphs. The printed struts are outlined with dashed lines for enhanced visibility.

To demonstrate that the produced fibers were suitable for bioprinting and that flow‐induced fiber alignment could be achieved, 15 µm diameter electrospun PCL fibers of varying lengths were incorporated into 30% Pluronic and bioprinted. As shown in **Figure** **9**, fibers of both lengths aligned successfully within the ink. This was achieved at a fiber concentration of 2% (w/v) without the formation of aggregates or clogging.

This highlights one of the key advantages of the processing method described here when integrated with bioprinting. Larger fibers can be reliably processed, and their lengths easily adjusted. By tuning parameters such as fiber stiffness, diameter, and length, aggregation and clogging during printing can be effectively managed. Our collection and cutting method allows for precise tailoring of these parameters, significantly accelerating the identification of optimal fibers for specific bioprinting applications. This is particularly notable when compared to other cutting or fragmentation methods, such as those described in the introduction, which are often limited to specific materials or processing conditions. Furthermore, the quantities of fibers produced using our method are sufficient for experimental applications and comparative studies.

Another important consideration is fiber length distribution. A wider length distribution typically results in more long fibers within a batch, which increases the likelihood of entanglement and aggregation during printing. The relatively low standard deviation of 50 µm and narrow length distribution achieved with our method offer a distinct advantage. This ensures that the fibers can be incorporated more homogeneously into the ink, minimizing aggregation and entanglement. As a result, the bioprinting process is more consistent, with fewer instances of clogging and more reliable outcomes.

## 3 Conclusion

There is a growing interest in utilizing fibers as filler materials in hydrogels and bioinks for biofabrication and tissue engineering. These fibers mimic fibrillar structures such as collagen,^[^ ^6^ ^]^ stabilize the inks,^[^ ^77^, ^78^ ^]^ modulate rheological properties^[^ ^12^, ^31^ ^]^ and serve as guides for cell anisotropy and tissue alignment during maturation.^[^ ^14^, ^21^ ^]^ While some research has explored the use of fiber fragments, many questions remain unanswered regarding how cut fiber diameter, length, surface chemistry, stiffness, shape, and material influence fiber‐cell interactions and behavior. Additionally, the impact of these characteristics on hydrogel stability, printability, and fiber alignment during printing is still not fully understood. This lack of understanding is primarily due to the labor‐intensive nature of most fiber fragment production methods, which are often difficult to adapt to different materials or fiber sizes, leading to limited variability, low yields, or high fiber polydispersity.^[^ ^26^, ^78^ ^]^

In this study, we demonstrated the efficacy of the described fiber collection and cutting method, which employs cryo‐cutting in conjunction with fiber collection on soft adhesive PVA films. This approach stabilizes and facilitates the easy transfer of fibers. Our method can be seamlessly augmented and applied to various fiber production techniques and non‐water/ethanol‐soluble fibers with diameters ranging from the nanoscale to above 100 µm. It results in pure cut fibers of precisely variable lengths and low polydispersity immediately after cutting, regardless of the fiber material, without necessitating changes to the cutting and purification process. This allows for the processing of a wide range of fibers, including both plastic and proteinaceous materials, that were previously difficult to transfer from the collector or cut. Most existing fiber spinning protocols can be easily adapted to incorporate this method, unlocking the potential to consistently harness appropriate amounts of micrometer‐long fibers for diverse applications. This versatility is not only relevant for biomaterial‐based applications but also for catalysis, where small cut fibers can function similarly to catalytic particles or substrates for binding substances.^[^ ^62^, ^79^, ^80^ ^]^ However, the primary focus of this work was to develop a reliable fiber processing method capable of producing distinct fibers of various sizes and materials from different spinning techniques for applications in cell culture, biofabrication, and tissue engineering. This was successfully demonstrated by producing a variety of fibers compatible with human fibroblast cells in vitro, showing no signs of cytotoxicity due to processing residues. Furthermore, the fibers could be coated or chemically modified without issue, enabling modulation of cellular behavior.

The developed processing technique for obtaining cut fibers significantly expands the possibilities for nano/micro‐sized cut fibers, allowing for effortless integration of different spinning methods, materials, and sizes. This enables comparisons and analyses of fiber fragments in biofabrication applications,^[^ ^33^ ^]^ such as fillers to enhance the stability of bioinks, cell guidance structures via flow‐induced alignement during extrusion bioprinting, and vessels for drug or factor release.

## 4 Experimental Section

### Materials

If not stated otherwise, all chemicals were supplied by Carl Roth GmbH + Co. KG Karlsruhe, Germany in analytical grade.

### PVA Film Preparation

Polyvinyl alcohol (PVA) films were prepared by casting a solution containing 130 mg mL^−1^ PVA (9000–12 000 Mn, Merck KGaA, Darmstadt, Germany), 15% (w/v) ethanol, and the desired concentration of glycerin, typically 11% (v/v). The films were cast onto either aluminum foil or metal plates (0.06 mL cm^−^^2^) that had been thoroughly cleaned with isopropanol. To stabilize the solution coverage during evaporation, thin FDM 3D printed frames were used as borders for the casting compartment, if needed. A glass slide was employed to distribute the solution evenly across the desired surface. The films were then dried under a fume hood for 1 day and stored at 20 °C with 40% humidity. For application, the films were used as‐is or, if a FDM frame had been employed, it was removed beforehand.

### Collagen Fiber Production by Microfluidic Wet Spinning

Collagen fibers were produced by wet spinning a 5 mg mL^−1^ rat tail collagen type I solution (Corning, New York, USA), which was diluted from an initial concentration of 8 mg mL^−1^ using acetic acid (pH 3). The solution was filtered through a 0.45 µm filter (Sarstedt, Numbrecht, Germany) and applied to the middle channel of a five‐channel microfluidic chip at a flow rate of 200 µL h^−1^. The side channels were flooded with the filtered precipitation buffer at flow rates of 400 µL h^−1^ for the outermost channels and 100 µL h^−1^ for the inner channels. The precipitation buffer contained 10% (w/v) polyethylene glycol 20 000, 4.14 mg mL^−1^ NaH2PO4·2H2O, 12.1 mg mL^−1^ Na2HPO4, 7.89 mg mL^−1^ NaCl, and 6.86 mg mL^−1^ HEPES, adjusted to pH 8 using NaOH.

The spun fiber was extruded into the precipitation buffer bath and collected onto a tube covered with soft PVA foil. After collection, the foil was removed, and the fibers were cryo‐cut to ≈100 and 300 µm lengths. The collected fiber pieces were washed twice in PBS and passed through a 70 µm pore size cell strainer (Falcon, Corning, New York, USA) to remove larger impurities and aggregates resulting from the spinning process.

### Melt–Electrowritten (MEW) PCL Microfiber Production

PCL fibers with diameters of ≈2, 15, 50, and 130 µm were produced using MEW. The printing process was conducted at 20 °C with 40% humidity, utilizing PCL (Purac PC12, Corbion, Amsterdam, the Netherlands) at a melt temperature of 95 °C. The diameters of the fibers were adjusted by varying the collector speed and extrusion pressure. Fibers were printed onto a grounded steel build plate that was covered with a thin layer of PVA film, with a voltage difference of 2.5 kV applied across a 22 G needle (Nordson EFD, Ebensfeld, Germany), maintained at a printing distance of 2.4 mm.

### Electrospinning of PCL and PLA Fibers

PCL and PLA fibers were produced by applying a voltage difference of 9.5 kV to a 23 G needle (Microlance BD, New Jersey, USA). A solution of 300 µl containing 12% (w/v) polycaprolactone (80 kDa, Sigma Aldrich, MO, USA) or 12% (w/v) PLA (100 kDa, Sigma Aldrich, MO, USA) in 99% pure 1,1,1,3,3,3‐hexafluoro‐2‐propanol (HFIP, abcr GmbH, Germany) was spun at a rate of 3 mL h^−1^, with a distance of 17 cm between the needle and collector. The grounded rotating drum collector (Ø 94 mm) was rotated at a speed of 1600 rpm to facilitate fiber alignment. The fibers were deposited onto aluminum foil that was thinly coated with soft PVA film attached to the collector.

### Gelatine Fiber Production

Gelatin fibers were produced using a voltage difference of 15 kV applied to a 23 G needle (Microlance BD, New Jersey, USA). A solution containing 300 µl of 10% (w/v) gelatin (≈300 g Bloom, Type A, Merck KGaA, Darmstadt, Germany) in 99% pure 1,1,1,3,3,3‐hexafluoro‐2‐propanol (HFIP, abcr GmbH, Germany) was spun at a rate of 3 mL h^−1^, maintaining a distance of 17 cm between the needle and the collector. The grounded rotating drum collector (Ø 94 mm), equipped with rods spaced 3 cm apart, was rotated at a speed of 1600 rpm to promote fiber alignment. After spinning, the fibers were collected onto a strip of soft PVA film by suspending it on a support frame. The film was inserted through the side of the collector and pulled outward from behind the fibers, allowing for immobilization of the fibers on the film. The unfolded films containing the fibers were then placed in a petri dish and crosslinked using glutaraldehyde vapors for 24 h. Following crosslinking, the glutaraldehyde was allowed to evaporate, and the films were further processed as outlined in the cutting procedure.

### Fiber Cutting Procedure

The films with the collected fibers on top were cut into strips perpendicular to the fiber alignment. The strips were then carefully removed from the metal plate or aluminum foil by pulling them off and subsequently rolled up. Several of these rolls were pressed together and immersed in a tube containing Tissue‐Tek O.C.T. compound (Sakura Finetek, Tokyo, Japan), which was then rapidly frozen in liquid nitrogen and stored at −20 °C. The collected fibers were cryo‐cut using a cryotome (Leica, CM3050 S) at −20 °C. The cutting length could be adjusted on the cryotome, ranging from 10 to 300 µm in various increments. The cut discs were collected, and the water‐soluble foil/Tissue‐Tek O.C.T. was removed by washing in 70% ethanol three times for fibers made from PCL or PLA. In contrast, gelatin and collagen fibers were washed in PBS. For storage and to determine their mass, the fibers were transferred to H₂O and freeze‐dried.

### Gelatine Coating and Fluorescence Modification

The gelatin coating process involved first activating the MEW PCL fibers in 100 mm NaOH for 30 min. The fiber suspension was then mixed 1:1 v/v with a solution of 0.5 mg mL^−1^ gelatin (≈300 g Bloom, Type A, Merck KGaA, Darmstadt, Germany) in 120 mm HEPES buffer, pH 7, and incubated for 45 min. After incubation, the fibers were pelleted by centrifugation and thoroughly washed with 20 mm HEPES buffer. The washed fibers were then resuspended in 20 mm HEPES and modified with 50 µM NHS‐Fluorescein (Merck KGaA, Darmstadt, Germany) for 15 h. Finally, the fibers were washed again with 20 mm HEPES and deionized water before being freeze‐dried.

### Tensile Testing

Tensile testing of the different reinforcement rings and scaffolds was performed using a universal testing machine (Z010, Zwick Roell, Ulm, Germany) equipped with a 100 N load cell. Samples were mounted between two clamps and stretched at a velocity of 10 mm min^−1^, with the upper force limit set to 95 N. The force exerted on the samples was measured as a function of deformation, and the results were evaluated based on a surface area of 100 µm × 1 cm.

### Film Adhesion Testing

A dynamic mechanical testing device (ElectronForce 5500, TA Instruments) was used to measure the exerted force on a 0.5 cm^2^ required to detach it from a horizontally aligned metal plate.

### Fourier Transform Infrared Spectroscopy (FTIR) Measurements

The samples were measured dry using a Nicolet iS10 with smart iTR diamond ATR (attenuated total reflectance, Thermo Fisher Scientific, Waltham, USA).

### Thermogravimetric (TGA) Measurements

TGA was measured with a TGA 2 (Columbus, USA). The samples of ≈4 mg were heated from 25 to 640 °C at a heat rate of 10 K min^−1^ and under constant nitrogen flow (50 mL min^−1^).

### Scanning Electron Microscopy (SEM)

To evaluate fiber length and diameter, the collected fibers were immersed in 70% ethanol or water and transferred onto SEM sample holders using a pipette. After drying, the fibers were sputter‐coated with 4 nm of platinum using an EM ACE600 sputter coater (Leica). Images were then captured with a Crossbeam CB 340 SEM (Carl Zeiss), and the lengths and diameters of the fibers were measured using ImageJ software.

### Fiber Sterilization for Cell Culture

MEW PCL fibers measuring 2 × 50 and 15 × 100 µm were sterilized using 70% ethanol for 20 min. In contrast, the electrospun gelatin fibers (3 × 50 µm) and gelatin‐coated PCL fibers were sterilized using UV light at 265 nm for 20 min.

### Cell Viability Staining with BJ Fibroblasts

BJ‐TERT human fibroblasts were cultured at a density of 4000 cells per 0.1 mg of the specified fiber type (2 × 50 and 15 × 100 µm MEW PCL, and 3 × 50 µm electrospun gelatin) in DMEM high‐glucose medium (Thermo Fisher Scientific, Waltham, USA) supplemented with 10% fetal calf serum and 5000 U mL^−1^ penicillin‐streptomycin (both from Thermo Fisher Scientific, Waltham, USA).

Viability staining was performed on days 1, 4, and 7 using the Live/Dead staining kit (L3224, Invitrogen, Thermo Fisher Scientific, Waltham, USA). Samples were incubated with 2 µm Calcein acetoxymethyl ester and 2 µm ethidium homodimer‐1 in phosphate‐buffered saline (PBS) for 30 min at 37 °C, followed by immediate imaging with a fluorescence microscope (Axio Observer, Zeiss, equipped with epifluorescence optics and a MRm camera; Zeiss, Oberkochen, Germany). Cell viability was assessed by counting living and dead cells from one overview image of each sample (n = 3) for every group, and the results were presented in bar charts.

### Confocal Microscopy of BJ‐TERT Tomato Fibroblasts on Fibers

TdTomatofarnesyl‐expressing BJ‐TERT human fibroblasts were generated as previously described.^[^ ^69^ ^]^ and utilized for immunofluorescent analysis. These fibroblasts were cultured at a density of 30 000 cells per 0.1 mg of the specified fiber type (2 × 50 and 15 × 100 µm MEW PCL, with and without a fluorescently labeled gelatin coating, as well as 3 × 50 µm electrospun gelatin fibers that were also fluorescently labeled) in DMEM high‐glucose medium (Thermo Fisher Scientific, Waltham, USA) supplemented with 10% fetal calf serum and 5000 U mL^−1^ penicillin‐streptomycin (both from Thermo Fisher Scientific, Waltham, USA). Cell‐fiber interactions were imaged using confocal microscopy (TCS SP8, Leica, Wetzlar, Germany) after one day of culture.

### Extrusion Bioprinting with Fibers

Pluronic F‐127 (KGaA, Darmstadt, Germany) at a concentration of 30% (w/v) in H_2_O was mixed with MEW PCL fibers of sizes 15 × 100 or 15 × 300 µm at a fiber concentration of 20 mg mL^−1^. Before incorporation, the lyophilized fiber pellets were resuspended in 70% ethanol and washed with water to remove the ethanol. The cooled Pluronic hydrogel was then added to the pellet and the fibers mixed into the gel using a spatula. The mixture was printed using an Inkredible+ bioprinter (Cellink Bioprinting AB, Gothenburg, Sweden) equipped with a 20G conical nozzle (Nordson EFD, Ohio, USA) at a temperature of 21 °C and an extrusion pressure of 22 kPa. The samples were printed in a line pattern onto a glass slide at a printing speed of 5 mm s^−1^.

### Statistical Analysis

Statistical analyses were conducted using a two‐way analysis of variance (ANOVA) with OriginPro software (OriginLab, Northampton, USA) to assess statistical significance. Tests for normality and homogeneity of variance were performed prior to analysis. Differences were considered statistically significant at p‐values below 0.05.

## Conflict of Interest

The authors declare no conflict of interest.

## Supporting information



Supporting Information

## Data Availability

The data that support the findings of this study are available in the supplementary material of this article.
