# Supplementary material for: A Versatile Method to Produce Monomodal Nano‐ to Micro‐Fiber Fragments as Fillers for Biofabrication
Source: Small Methods. 2024 Dec 17;9(3):2401060. doi: 10.1002/smtd.202401060 (PMC11926501; doi:10.1002/smtd.202401060)
Supplement: Supplementary file 1 — Supporting Information [file SMTD-9-2401060-s001.docx]

Supporting Information

**A Versatile Method to Produce Monomodal Nano- to Micro-Fiber Fragments as Fillers for Biofabrication**

Zan Lamberger, Vivien Priebe, Matthias Ryma, and Gregor Lang*


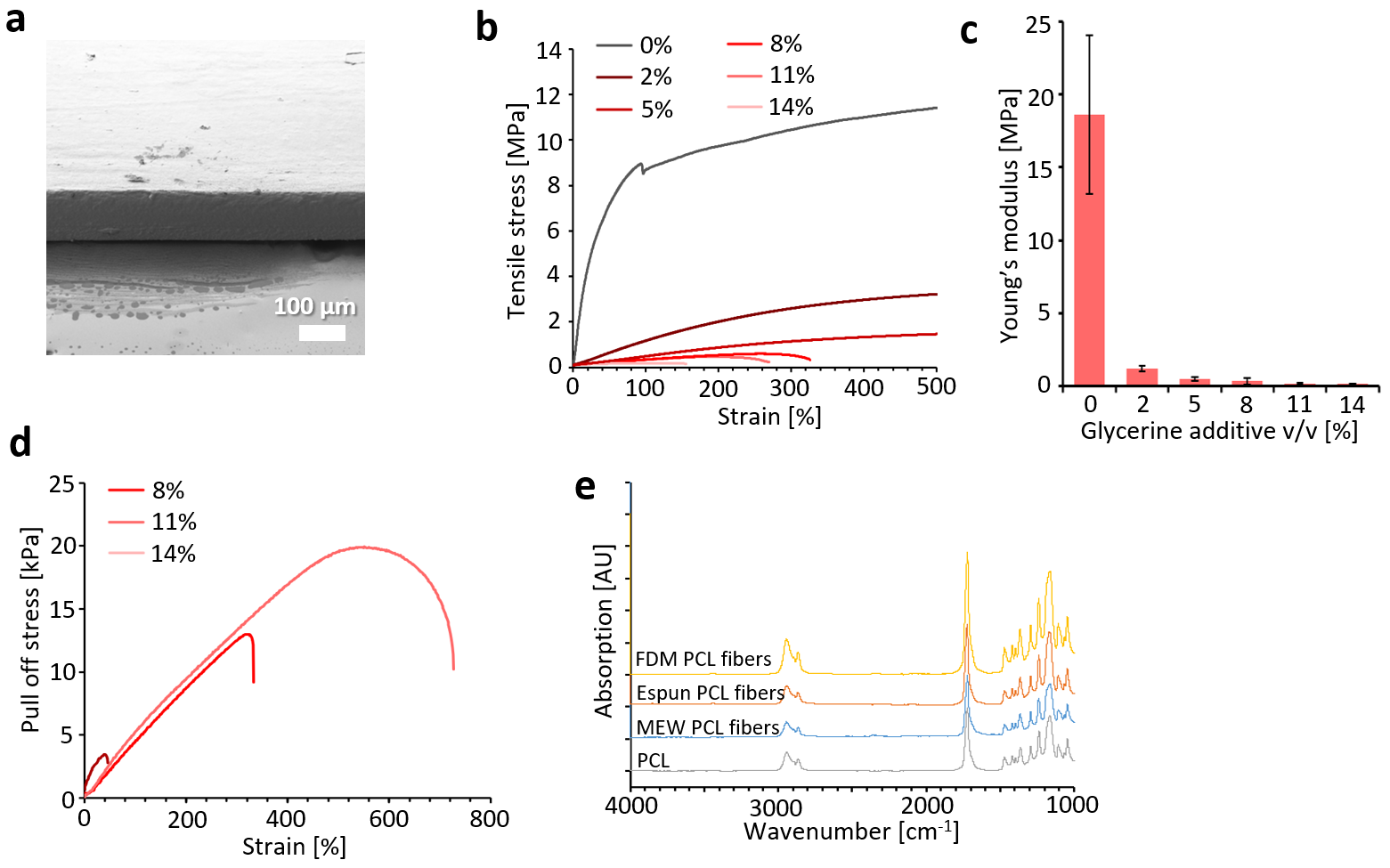


**Figure S1.** a) Cross-section of the PVA film used to determine an approximate height of 100 µm. b) Representative tensile testing curves for PVA films with varying glycerin additive concentrations. c) Young’s modulus derived from tensile testing of films supplemented with different glycerin amounts. d) Representative adhesion test curves for films with increasing glycerin additive concentrations. e) Shows the FTIR spectra of PCL fibers after being cut and purified but produced using different spinning techniques. The plotted values in c) represent the mean ± standard deviation of Young’s modulus (n=9).


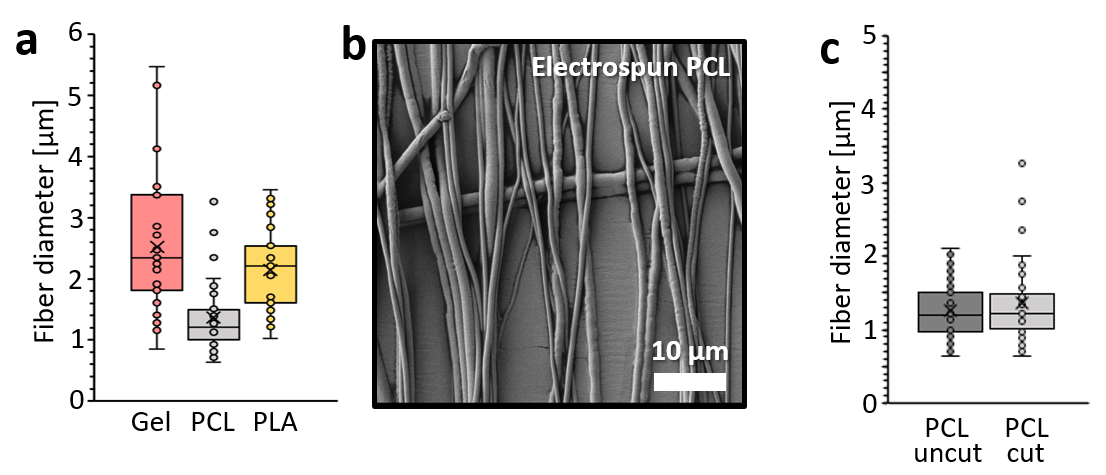


**Figure S2.** a) Diameters of the fibers shown in **Figure 4**. b) Exemplary image of an electrospun PCL membrane on the film. c) The diameters of the electrospun PCL fibers before and after undergoing the cutting procedure. The plotted values represent the mean ± standard deviation of fiber diameters, with "x" indicating the average and "–" indicating the median. Sample sizes are as follows: for a) Gelatin (n=35), PCL (n=50), PLA (n=50), for c) n=50.


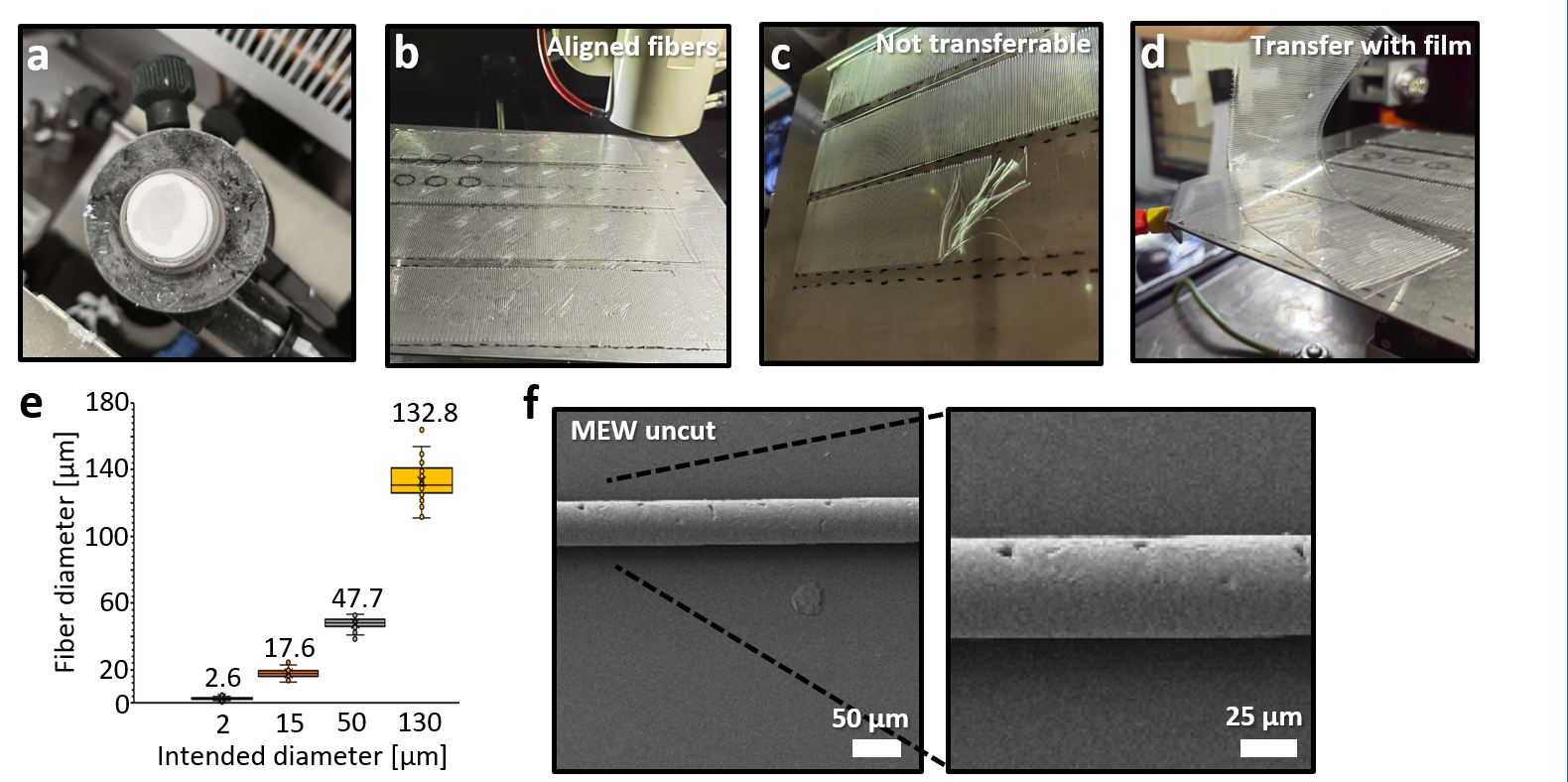


**Figure S3.** a) Rolled-up PVA foils mounted in the cryo cutter after being cut. b) Production of aligned fiber mats using MEW. c) Direct transfer of fibers from the print bed leads to misalignment and entanglement. d) Seamless transfer is achieved by depositing fibers onto a PVA film. e) Diameters of the printed fibers as shown in **Figure 5**. f) Image of a melt electrowritten 50 µm diameter fiber before cutting. The plotted values represent the mean ± standard deviation of fiber diameters, with "x" indicating the average and "–" indicating the median. Sample sizes: 2 µm (n=50), 15 µm (n=30), 50 µm (n=50), 130 µm (n=50).


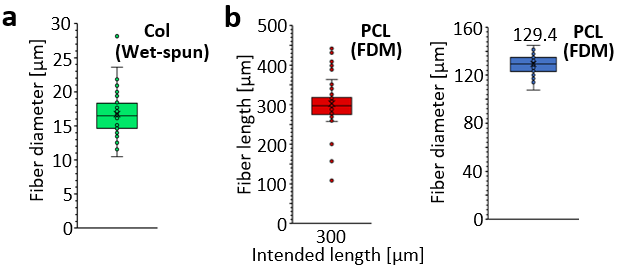


**Figure S4.** a) Diameters of the collagen fibers shown in **Figure 5**. b) Fiber length and diameter measurements for the FDM-printed cut fibers. The plotted values in a) represent the mean ± standard deviation of fiber diameters, with "x" indicating the average and "–" indicating the median (n=36). The plotted values in b) represent the mean ± standard deviation of fiber length and diameters, with "x" as the average and "–" as the median (n=50 for both length and diameter).


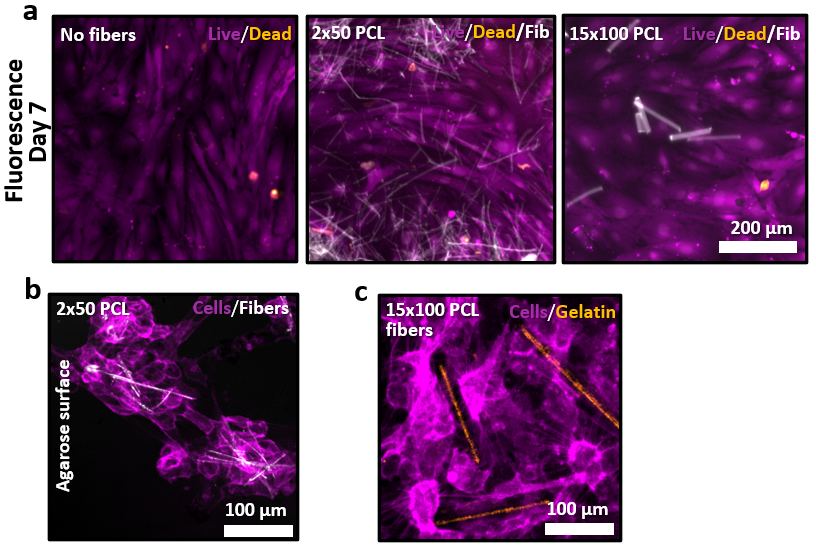


**Figure S5.** a) Representative live-dead images taken on day 7 of BJ fibroblast culture without fibers, and with 2x50 and 15x100 PCL fibers, highlighting the autofluorescence of the fibers. b) Autofluorescent BJ cells interacting with uncoated PCL fibers when cultured on agarose. c) Autofluorescent fibroblasts surrounding gelatin-coated 15x100 PCL fibers, with reduced fiber fluorescence to better illustrate the cellular interaction.
